# Current status of cytokine-induced killer cells and combination regimens in breast cancer

**DOI:** 10.3389/fimmu.2025.1476644

**Published:** 2025-02-06

**Authors:** Yuancong Jiang, Jie Qiu, Nanwei Ye, Yingchun Xu

**Affiliations:** ^1^ Department of Breast and Thyroid Surgery, Shaoxing People’s Hospital, Shaoxing, China; ^2^ Department of Medical Research Center, Shaoxing People’s Hospital, Shaoxing, China

**Keywords:** breast cancer, cytokine-induced killer cells, combination therapies, immunotherapy, molecular targeted therapy

## Abstract

Breast cancer remains a significant health challenge worldwide, with substantial efforts aimed at understanding its pathogenesis, biological characteristics, and clinical triggers. Recently, immunotherapy such as the cytokine-induced killer cells combined with other drug therapies has offered new hope for patients with advanced breast cancer. However, the specific pathogenesis of combination regimens involving cytokine-induced killer cells remains elusive. Besides, the combination of immunotherapy with cytokine-induced killer cells might represent a novel breakthrough. This review outlines the current status of cytokine-induced killer cell therapies and their combination strategies, especially the combination of chemotherapy with molecularly targeted treatments, for the management of breast cancer.

## Introduction

As is well documented, breast cancer is one of the most prevalent cancers among women worldwide that poses a major threat to their health (1). According to the latest data released by the World Health Organization in 2020, approximately 2.2614 million new cases of female breast cancer patients were diagnosed worldwide, accounting for 11.7% of all cancer cases globally and ranking first among all malignancies. Meanwhile, breast cancer resulted in approximately 685,000 deaths, accounting for 6.9% of all cancer-related deaths globally and ranking fourth among all malignant tumor-related deaths (2). Similarly, the latest statistics on cancer incidence in China revealed that in 2013, roughly 278,800 individuals were diagnosed with breast cancer, with about 64,600 dying of the condition, which seriously threatens human life and health (3, 4). Despite efforts to address the severe burden of breast cancer, traditional treatment strategies, such as chemotherapy, have resulted in limited breakthroughs and failed to deliver satisfactory therapeutic effects due to the unique biological behavior and clinical characteristics of breast cancer (5, 6).

Cytokine-induced killer (CIK) cells are a population of heterogeneous immune effectors, primarily composed of CD3+ and CD56+ T cells, induced by a combination of cytokines (IFN-γ, IL-1, IL-2, etc.) and anti-CD3 antibodies. They share phenotypical and functional characteristics with both NK and T cells (7, 8) and exert anti-tumorigenic effects by promoting the expression of MHC-I molecules on tumor cells and increasing the killing capabilities of the cytotoxic T cells (CTL) (9). Recent research indicated that the release of IFN-γ by CIK cells positively impacts CTL apoptosis induction (10). Moreover, apoptosis-related genes of tumor cells can also be activated by CIK cells (11). In contrast to cascade-primed immune cells (CAPRI), another prominent adoptive cell immunotherapy with specific cytotoxic effects on tumor cells, CIK cells exert strong non‐specific cytotoxic activity (12). Moreover, compared with another type of cytotoxic effector T cells, lymphokine-activated killer (LAK), CIK cells demonstrate higher tumor cell lytic activity and proliferation rates, as well as lower toxicity (13). As a novel type of adoptive immunotherapy approach, CIK therapy can exert favorable therapeutic effects for patients resistant to traditional therapies.

Indeed, CIK therapy promotes intracellular adhesion and combination by expressing CD56 molecules and can release perforin, cytolysin, etc., to induce osmotic dissolution of target cells, is not likely to elicit graft-versus-host disease, and typically causes only mild adverse reactions such as fever (14, 15). For patients with multi-drug resistance who have failed chemotherapy, CIK therapy can also yield significant outcomes. Although it is generally recognized that the tumor microenvironment (TME) and immune evasion play a pivotal role in the progression of breast cancer, effective treatments to overcome the immunosuppressive effects of cancer-related TME remain limited. CIK therapy has emerged as a novel therapeutic method and has achieved promising results to compensate for the shortcomings of traditional therapies. Numerous studies have established that CIK therapy is an effective method for the treatment of malignant solid tumors. For instance, the combination of CIKs with dendritic cell (DC) has been applied in cancer immunotherapy and can significantly drive IL-12 secretion and enhance the cytotoxic activity of CIKs (16). CIKs are usually combined with other treatments rather than used alone, given that some chemotherapies can stimulate tumor-specific T cells, and subsequent CIK therapy can further enhance tumor-specific immune responses (17, 18). Therefore, CIK cells combined with chemotherapy can play a synergistic role.

Despite the progress in targeted therapy for cancer, CIK therapy remains rarely applied in clinical practice. According to studies, the combination of anlotinib and murine CIKs improved CD3+ T cell and CD3+CD4+ T cell infiltration and up-regulated the expression of granzyme B and IFN-γ in CD3+CD8+ T cells, thereby increasing antitumorigenic activity (19). CIK activity can be further combined with monoclonal antibodies (mAbs), considering that CIKs express CD16 in a donor-dependent manner and foster potent antibody-dependent cell-mediated cytotoxicity (ADCC) (20). Of note, ADCC plays an essential role in mediating the antitumorigenic effects of routinely used therapeutic mAbs such as trastuzumab and cetuximab, which are approved for the treatment of HER2+ and EGFR+ tumors, such as breast, gastric, and colorectal cancers (21).

This review aimed to summarize the current status and challenges in the development of CIK therapy and primarily focused on combination strategies, especially chemotherapy and molecularly targeted treatments, for breast cancer.

## Production and mechanisms underlying cytokine-induced killer cell therapy

CIK cells are originally derived and isolated from patient peripheral blood mononuclear cells (PBMCs) via density-gradient centrifugation. In 1991, Schmidt-Wolf et al. first described CIKs using C.B-17 severe combined immune deficient (SCID) mice as an experimental model. By incubating human PBMCs, rIFN-γ (1000 U/mL) was initially added on day 0 of culture. On the second day, mAb anti-CD3 (50 ng/mL), IL-2 (300 U/mL), and human rIL-1 (100 U/mL) were added. Then, fresh IL-2 was added during culture, effectively expanding CIK cells *in vitro* (22). The extracted CIK cell population mainly comprises CD3+ heterogeneous T cells, with approximately 40%-80% of positive CD3+CD56+ NK-like T cells, 20-60% of negative CD3+CD56− T cells, and a small subpopulation of CD3-CD56+ NK cells accounting for less than 10% of the total cell population. The anti-tumorigenic effect is predominantly attributed to CD3+CD56+ NK-like T cells. In addition, other cells, such as CD4+T cells, CD8+T cells, and T regulatory lymphocytes (Treg cells), may be present in the CIK cell population, which also affects the efficacy of immunotherapy (23, 24). According to an earlier report, the proportion of CIKs varies across studies. **Table 1** displays the subgroups of cytokine-induced killer cell therapy used in clinical trials. For instance, Palmerini et al. employed G-Rex devices and stimulated G-Rex with clinical-grade IFN-γ, anti-CD3 antibody, and IL-2 to produce a large number of CIKs. CIKs generated in G-Rex exhibited a less differentiated phenotype and robust expansion ability that reduced cultivation time and cost for *in vitro* CIK preparation (29). In addition, previous studies have uncovered that as recognition structures of CIKs, natural killer group 2D (NKG2D) engagement alone could induce degranulation, IFN-γ secretion, and lymphocyte function-associated antigen (LFA-1) activation on mature CIK cells through similar signaling pathways (PI3K, PLC-γ, and Src) to those observed in NK cells (30). Notably, NKG2D is considered a key contributor to the MHC-unrestricted cytolysis of CIK cells against various types of tumors (31). Consequently, enhancing the targeted NKG2D axis is a promising method for improving CIK therapy in cancers.

**Table 1:** Subgroups of cytokine-induced killer cell therapy in clinical trials.

Reference	CD3^+^ cells	CD3^+^ CD4^+^ cells	CD3^+^ CD8^+^ cells	CD3^-^ CD56^+^ cells	CD3^+^ CD56^+^ cells	CD16^+^ CD56^+^ cells	CD19^+^ cells
Lin et al., 2017 (25)	/	11%–13%	79%–82%	/	/	3%–5%	2%–4%
Pan et al., 2014 (26)	80-90%	/	60-80%	6-22%	/	/	/
Yang et al., 2022 (27)	97.75% (43.1-99.5%)	24.3% (1.7-74.3%)	70.35% (17-93.3%)	1.6% (0.2-55.7%)	21.2% (4.3-66.3%)	/	/
Zhou et al., 2019 (28)	75.9-93.4%	15.3%-21.3%	40.1%-80.3%	4.5-11.1%	6.1-57.9%	/	/

Prof. Schmidt-Wolf’s teams and others have demonstrated that CIK cells are compatible with almost all types of immune checkpoint inhibitors, epigenetics drugs, and commercial compounds. With the advent of contemporary techniques such as CAR-CIK therapy, combination strategies involving CIKs are currently being explored for cancer treatment. Magnani et al. employed donor-derived CD19 CAR T cells generated with the Sleeping Beauty (SB) transposon and differentiated into CIKs for patients with B cell acute lymphoblastic leukemia (B-ALL) who relapsed after allogeneic hematopoietic stem cell transplantation (HSCT), which achieved antileukemic activity without eliciting severe toxicities (NCT03389035) (32). Zhou et al. reported the application of a combined strategy involving CIKs and programmed cell death-1 (PD-1) inhibitors in stages IIIB-IV non-small cell lung cancer patients, showing promising results (NCT03987867) (33). Similarly, Liu et al. observed that a reasonable sequence of pemetrexed combined with CIK therapy and anti-PD-1 mAbs significantly promotes the efficacy of CIK therapy in NSCLC (34). Zhou et al. identified PD-L1 expression in breast cancer as an indicator of adjuvant CIK therapy in patients with postoperative breast cancer. Interestingly, higher PD-L1 expression in the CIK group was associated with longer overall survival (OS) and recurrence free survival (28). Furthermore, a higher number of CIK treatment cycles resulted in longer disease-free survival (DFS) and OS in triple-negative breast cancer (TNBC) patients, and the majority of patients who benefitted from CIK therapy were those with relatively early-stage TNBC (13). Sommaggio et al. established the primary and metastatic model of breast cancer by implanting TNBC samples from patients in NSG mice or intravenously injecting MDA-MB-231 cells, followed by the intratumoral or intravenous injection of CIKs and cetuximab to explore the potential of this new combined strategy (7). While most studies focused on combination strategies for the treatment of TNBC, the efficacy of CIK therapy in various types of advanced breast cancer remains elusive and warrants further exploration.

**Figure 1:**
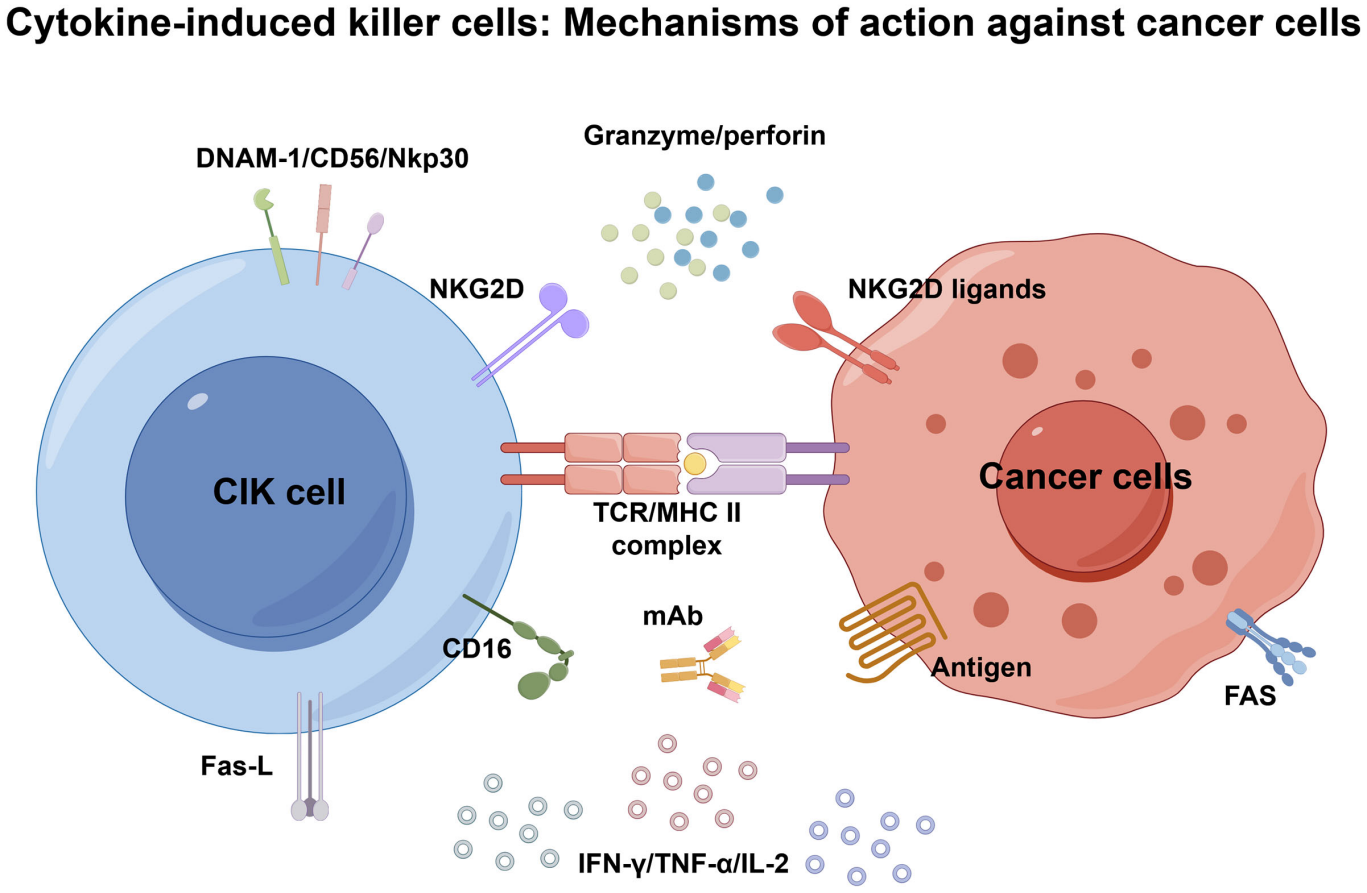
Mechanisms of action of CIK cells against cancer.

As reported in breast cancer research, CIK can recognize tumor cells using polyclonal T cell receptors (TCR) in a classical MHC-restricted manner (24). In addition, CIK cell cytotoxicity can be exerted against various hematological and solid tumors through the engagement of the NKG2D, enabling MHC-unrestricted tumor recognition without prior exposure to antigens or priming (8). This MHC-independent antitumor activity was mostly mediated by CD3+CD56+ NK-like T cells, which release toxic particles (such as granzyme and perforin) that target hematological and solid malignancies while sparing healthy tissues or hematopoietic precursors (20). In addition, natural killer receptors such as DNAM-1, CD56, and Nkp30 also contribute to the MHC-unrestricted tumor recognition capabilities of CIK cells, which further enhance their cytotoxicity. Furthermore, CIK cells secrete numerous cytokines, encompassing IFN-γ, TNF-α, and IL-2, to induce anti-tumor immune responses in patients and participate in the regulation of innate and adaptive immunity (35). Some studies documented that the anti-tumor effect of CIK cells is related to the down-regulated expression of the c-Myc gene and the induction of apoptosis via the expression of Fas-L (28, 36). The mechanisms of CIK cell-mediated anticancer activity are illustrated in **Figure 1**.

## Combination treatment with dendritic cell immunotherapy

At present, while CIK therapy is widely used for the treatment of hematological tumors and solid tumors, such as liver cancer, clinical studies investigating its application in breast cancer remain scarce (8). Compared with traditional treatments, CIK therapy generally requires a combination with other treatment options to achieve synergistic therapeutic effects. A common combination regimen is CIK with DC immunotherapy. In a retrospective observational study, patients with stage IV breast cancer treated with the DC vaccine and CIK cell therapy following chemotherapy experienced significantly longer DFS and OS compared with those who underwent chemotherapy alone (5-year DFS and OS: 42% and 44% vs 30% and 29%, respectively) (25). The infusion of DCs and CIKs, compared with CIK alone, irrespective of chemotherapy or not, was also reported to exert beneficial effects and marginal side effects in patients across four independent clinical trials (37–40). A meta-analysis of 11 relevant studies involving 941 patients also demonstrated consistent results, with significantly higher complete response (RR = 1.54, 95% CI: 1.09–2.19), partial response (RR = 1.33, 95% CI: 1.11– 1.59) and overall response rates (RR=1.37, 95% CI: 1.20–1.57) in breast cancer patients treated with DC+CIK+ chemotherapy regimen compared to those who underwent chemotherapy alone; moreover, the frequency of adverse events was comparable between these groups (41). Besides, a pre-clinical trial demonstrated that the DC vaccine and CIK cell treatment enhanced the cytotoxic efficacy of CIK cells activated by DCs sensitized with anchored HER polypeptide antigen on MCF-7 cells compared to CIK cells alone and that this combination could be used to develop a therapeutic DC vaccine for breast cancer (42).

## Combination treatment with chemotherapy

Chemotherapy is a traditional method for the treatment of breast cancer, especially for TNBC, which lacks the expression of estrogen and progesterone receptors and human epidermal growth factor receptor 2. However, chemotherapy is often limited by resistance and immune escape mechanisms, leading to suboptimal prognoses in patients with triple-negative breast cancer. A retrospective study enrolling 294 post-surgery TNBC patients concluded that the combination of chemotherapy with adjuvant CIK was associated with lower relapse and metastasis rates compared with chemotherapy alone and effectively prolonged survival time, especially in early-stage patients (13). Consistent with these findings, post-mastectomy TNBC patients with lymph node metastasis, advanced TNM stage, and poor pathological grades exhibited significantly higher DFS and OS rates in the chemotherapy+CIK treatment group compared with the chemotherapy group (26). A single-arm study investigated a regimen composed of cyclophosphamide, thiotepa, and carboplatin in combination with DC-CIK and reported a median PFS of 13.5 months, an OS of 15.2 months, and a mortality rate of 26.1% in 23 TNBC patients (43). Similarly, combination strategies incorporating CIKs are equally effective in the treatment of recurrent and metastatic TNBC. For instance, maintenance therapy with metronomic capecitabine chemotherapy combined with DC-CIK immunotherapy improved patients’ immune function, enhancing their quality of life and prolonging progression free survival (44). However, a study enrolling 340 patients with TNBC provided evidence for the prolonged OS of patients with TNBC in the N1, N2, and N3 stages with the addition of CIK treatment but identified no statistical difference in 5-year DFS between the CIK cell treatment after chemotherapy group and chemotherapy alone group (77.9% vs. 69.8%, p = 0.159). They also observed no significant differences in the rates of local recurrence, regional metastasis, and distant metastasis between the two groups (7.8% vs. 7.6%, p = 0.957; 6.5% vs. 4.6%, p = 0.494; and 13.0% vs. 16.3%, p = 0.643, respectively) (45). As previously mentioned, PD-L1 expression in tumor tissue can serve as an indicator of the efficacy of adjuvant CIK treatment in postoperative patients. In the CIK cell treatment group, patients with high PD-L1 expression displayed superior survival benefits compared to those with low PD-L1 expression (28). Nevertheless, these studies only included a limited number of patients and offered low-level evidence, with most focusing on TNBC. Therefore, additional randomized controlled studies are necessitated to examine the safety and effectiveness of the combination of chemotherapy and CIKs in other subtypes. Relevant clinical studies and their administration of CIK therapy in breast cancer are listed in **Table 2**.

**Table 2:** Clinical studies and their administration of CIK cell therapy in breast cancer.

Reference	Object or Stage	Treatment subgroup	CIK cells administration	Status
Lin et al., 2017 (25)	IV	188 vs 180 (DC+CIK+chemotherapy vs chemotherapy)	Cells were collected on four consecutive days, with 6×10^9^ to 10×10^9^ cells being collected each day. The treatment was repeated four times in a fortnight, which formed one cycle and started at 7-day intervals after chemotherapy.	Retro
Mao et al., 2015 (37)	IV	20 vs 0 (DC+CIK+chemotherapy vs chemotherapy)	On the third day after chemotherapy, 1×10^9^ CIK and 1×10^7^ DC were suspended with 0.9% normal saline and then the suspension was injected into patients within 1h eight times alternatively.	Retro
Ren et al., 2013 (40)	IV	87 vs 79 (DC+CIK+high-dose chemotherapy vs standard dose chemotherapy)	At least 4.5x10^6^/kg CD34^+^ was collected to support the subsequent two cycles of high-dose chemotherapy and DC/CIK induction. After *in vitro* culture for 10–14 days, DCs and CIKs were harvested and administered intravenously at every 4-day interval for three cycles starting 1 week before high-dose chemotherapy.	Retro
Zhang et al., 2019 (13)	TNBC	147 vs 147 (CIK+chemotherapy vs chemotherapy)	On day 15 and day 16 of each chemotherapy cycle, patients received an intravenous infusion of at least 5×10^9^ CIK cells.	Retro
Pan et al., 2014 (26)	TNBC	45 vs 45 (CIK+chemotherapy vs chemotherapy)	After detection, all numbers of CIK cells (ranging from 8.7×10^9^ to 1.2×10^10^) were infused back to patients. The patients will receive at least 4 cycles of CIK cell infusion with 2-week intervals between each cycle.	Retro
Li et al., 2018 (45)	TNBC	77 vs 263 (CIK+chemotherapy vs chemotherapy)	The CIK cells were harvested, and the median number of CIK cells was 7.4 × 10^9^ with a viability of greater than 95%. After several cycles of chemotherapy, the patients were given an infusion of CIK cells.	Retro
Wang et al., 2016 (43)	TNBC	23 vs 0 (CIK+chemotherapy vs chemotherapy)	CD34+ progenitor cells were re-infused on day 3 and G-CSF was administered at 5 ug/kg/day from day 4 until polymorphonuclear cells reached above 1.5x10^9^/L.	Prospective
Yang et al., 2022 (27)	I-IV	48 vs 59 vs 107 (CIK+NK+ traditional treatment vs CIK+ traditional treatment vs traditional treatment)	Blood was collected at week 0 and CIK cells were performed at week 2. The peripheral blood needed by week 4 was collected before infusion at week 2.	Retro
Zhou et al., 2019 (28)	I-IV	150 vs 160 (CIK+traditional treatment vs traditional treatment)	The harvested autologous CIK cells that were free of microbial contamination were transferred to the patients by intravenous infusion within 30 minutes. Patients generally received CIK cell infusions for at least 4 cycles, with a 2-week interval between every 2 cycles.	Retro

## Combination treatment with molecular targeted therapy

Molecular targeted therapy targets identified oncogenic targets or related pathways and is capable of specifically interacting with oncogenic sites to induce the specific death of tumor cells. To date, targeted therapy has played a decisive therapeutic role in liver cancer, colon cancer, and breast cancer (46, 47). Sommaggio et al. (7) reported that CIKs plus cetuximab significantly inhibited the growth of primary tumors in patient-derived tumor xenografts and MDA-MB -231 cell line models and suppressed the formation of experimental and spontaneous lung metastases in mice. In another pre-clinical study, a focal adhesion kinase (FAK) inhibitor enhanced immune responses following co-culture with CIK cells; inhibition of FAK regulated the cGAS-STING pathway and PD-L1 expression and increased the TNBC cell-killing ability of CIK cells (48). Moreover, Zhou et al. identified PD-L1 expression as an independent prognostic factor for postoperative CIK treatment (28). Noteworthily, compared with trastuzumab or cetuximab monotherapy, the introduction of CIKs in patients with TNBC contributed to the re-sensitization of resistant cells to therapy (20). Mechanistically, the cytotoxicity of CIK cells is highly dependent on cell contact. Molecularly targeted agents have significantly promoted the sensitivity and cytotoxicity of TNBC cells in contact with CIK cells by regulating immune-related genes. It is recommended that the CIK cells with low spontaneous cytotoxicity be combined with mAbs to enhance their killing efficacy (49). To maximize the accumulation of anticancer agents within tumors, Liang et al. designed nanoplatforms to synergistically inhibit tumor progression by combining photothermal therapy and photodynamic therapy with trastuzumab and immunotherapy mediated by CIK cells for activating the immune response and deliver a precise strike to SKBR-3 cells (50). In summary, these studies corroborate that CIK can be used as an adjunct to targeted therapy in breast cancer to eliminate tumor cells and effectively prevent tumor recurrence.

## Other combination strategies with CIK therapy

Cytokine-related immunotherapy in breast cancer has been actively investigated over the past two decades, such as IL-12-based therapies (51). Mounting evidence suggests that IL-12, a novel and promising anti-tumor cytokine, plays a vital role in maintaining tolerance and contributing to immunity via regulation of TH1 immune response, presentation of tumor antigens, and production of IFN-γ (52, 53). In a pre-clinical study, IL-12 was identified as an effective adjuvant agent to CIK therapy, achieving complete tumor remission in 75% of treated animals, exceeding the effects of either therapy alone (53). This can be attributed to the broad immunostimulatory effects of low-dose IL-12, including enhanced IFN-γ secretion, increased cytotoxicity, and T-cell proliferation (54). More importantly, IL-12 may be most effective in combination therapy where lower doses can be applied. In addition to IL-12, the exogenous cytokines IL-2 and IL-7 can also assist peripheral blood lymphocytes in producing CIKs (55). It is worthwhile emphasizing that IL-12 can upregulate the expression of autophagy-related protein light chain 3 (LC3) and induce autophagosome formation by inhibiting the PI3K/Akt signaling pathway and activating the AMPK signaling pathway, thereby exerting anti-tumorigenic activity in breast cancer (56). Another study noted that treatment-resistant tumors harbor specific TGF-beta-activated cancer-associated fibroblasts (CAF), which are associated with low IL-2 activity. Stroma-targeted stimulation of the IL2 pathway in these unresponsive tumors restores the anti-cancer efficiency of trastuzumab (57). Taken together, these findings indicate that CIKs, in combination with interleukin, maybe a candidate approach for breast cancer treatment. Natural killer (NK) cells, a subset of lymphocytes in the innate immune system, are capable of recognizing a wide range of tumor cells in the human body. They serve as both direct effector agents against cellular targets and participate in and maintain a multicellular immune response (58). In a long‐term retrospective study conducted by Yang et al. (27), patients treated with a combination of CIK and NK cell immunotherapy for breast cancer exhibited a higher overall survival rate than those treated with CIK or NK cells alone. Specifically, the 5-year and 10-year survival rates in the CIK+NK group were significantly higher by about 19.17% and 25.01% in the TNBC group compared to the No-TNBC group, with more pronounced benefits observed in patients aged over 50 years. However, the cytotoxicity of NK cells is MHC-dependent (59), and genetically modifying NK cells and obtaining a stable source remain challenges to be resolved. In addition, the high cost of expanding NK cells and extracting specific interleukins highlights the need for more economical methods to selectively obtain a large number of proliferating immune cells.

**Figure 2:**
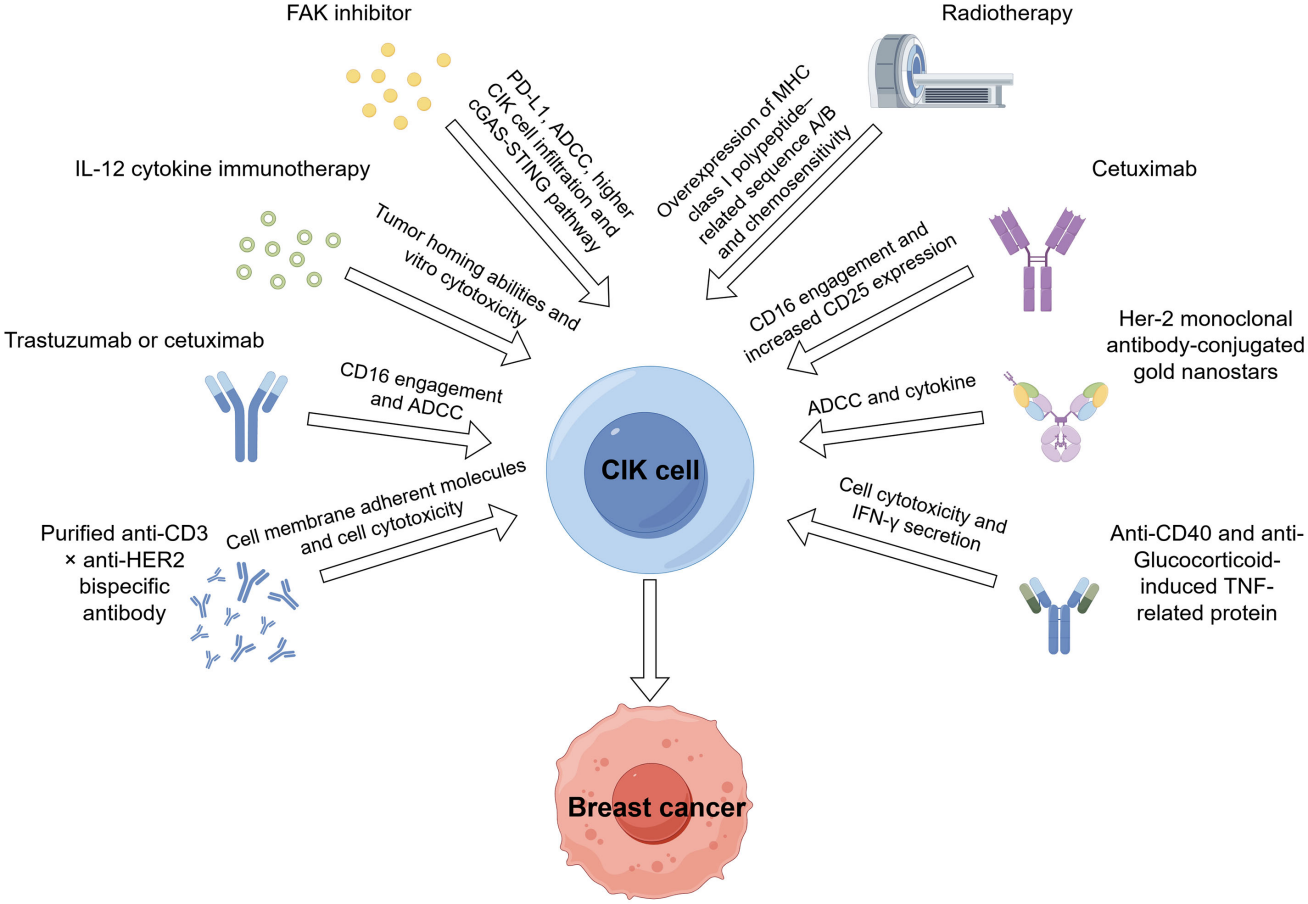
Mechanisms of action of chemotherapy, molecular target agents, and other combination strategies involving CIK cells in breast cancer.

Radiotherapy is one of the cornerstones of breast cancer treatment, especially for patients seeking breast conservation. However, the clinical response to radiotherapy is heterogeneous (60). Radioresistant affects the effect of radiotherapy. In a pre-clinical study, CIK cells enhanced the treatment of radioresistant breast cancer MCF-7 cells. Besides, the tumor cell recognition and cytotoxic activity of CIK cells are reliant on the interaction between NKG2D and class I-related molecules A and B (MHC A/B) and UL16-binding protein family members (ULBPs). Additionally, the secretion of IFN-γ and IL-6 by CIK cells also contributes to the tumor-killing ability of combination strategies involving CIK therapy and radiotherapy (24, 61). The potential therapeutic combinations involving cytokine-induced killer cells in pre-clinical studies are detailed in **Table 3**, whilst **Figure 2** delineates the mechanisms underlying chemotherapy, molecular targeting agents, and other combination strategies involving CIK cells in breast cancer.

**Table 3:** Potential therapeutic combinations with cytokine-induced killer cells in pre-clinical Studies.

Potential combinations agents	Target or pathway	Reference
Purified anti-CD3 × anti-HER2 bispecific antibody	Cell membrane adherent molecules and cell cytotoxicity	(49)
Trastuzumab or cetuximab	CD16 engagement and ADCC	(20)
IL-12 cytokine immunotherapy	Tumor homing abilities and vitro cytotoxicity	(53)
FAK inhibitor	PD-L1, ADCC, higher CIK cell infiltration, and cGAS-STING pathway	(48, 62)
Radiotherapy	Overexpression of MHC class I polypeptide–related sequence A/B and chemosensitivity	(61)
Cetuximab	CD16 engagement and increased CD25 expression	(7)
Her-2 monoclonal antibody-conjugated gold nanostars	ADCC and cytokine	(50)
Anti-CD40 and anti-Glucocorticoid-induced TNF-related protein	Cell cytotoxicity and IFN-γ secretion	(63)

ADCC, Antibody-dependent cell-mediated cytotoxicity; PD-L1, Programmed death-ligand 1; FAK, Focal adhesion kinase.

## Conclusive remarks

Overall, CIK cell therapy is a strong adjuvant antitumor approach in breast cancer. Results from several clinical trials and pre-clinical studies established that CIK cell therapy extends DFS and OS in patients with breast cancer and has the potential to be combined with molecularly targeted treatments to achieve more promising outcomes while minimizing side effects. Although the combination of CIKs with other drugs for treatment has been employed for many years, numerous limitations need to be addressed. Firstly, due to the nature of heterogeneous cells, challenges persist in optimizing production expansion and genetic modification to obtain a stable source of CIK cells (8, 64). Furthermore, the internal cytokine content of CIKs generated by *in vitro* stimulation varies, and their stability remains uncertain (8). Secondly, traditional cytotoxic drugs, targeted drugs, and endocrine drugs may result in peripheral blood test results or myelosuppression, posing challenges in capturing the specific reasons for changes in the content and composition of CIK cells in peripheral blood. For individuals developing myelosuppression following chemotherapy, the laborious and expensive production of effector cells poses an important obstacle to the implementation of combination strategies (7, 35). Notwithstanding, prospects for combination treatments involving CIK cell therapy are emerging and may be a promising therapeutic solution. Moreover, we speculate that some elderly patients, limited by multiple underlying diseases or with poor physical conditions, cannot tolerate surgical treatments and the cytotoxic effects of chemotherapy. Nevertheless, they can achieve long-term OS through CIK cell therapy alone or in combination with biological or targeted agents, with CIKs being a reliable treatment option.
